# The Surgical Treatment of Plantar Fibromatosis With Multiple Large Nodules on the Medial Aspect of the Left Sole With No Recurrence at the Five-Year Follow-Up

**DOI:** 10.7759/cureus.99349

**Published:** 2025-12-16

**Authors:** Christos Lyrtzis, Zoi Rafailia Sidiropoulou, Alexandra Arhonidu, Paissios Greige, Dimitrios Chytas, George Paraskevas

**Affiliations:** 1 Anatomy and Surgical Anatomy, Aristotle University of Thessaloniki, Thessaloniki, GRC; 2 Anatomy, Faculty of Health Sciences, Aristotle University of Thessaloniki, Thessaloniki, GRC; 3 Physiotherapy, University of Peloponnese, Sparta, GRC; 4 Anatomy and Surgical Anatomy, Faculty of Health Sciences, Aristotle University of Thessaloniki, Thessaloniki, GRC

**Keywords:** ledderhose disease, partial fasciectomy, plantar fibromatosis, reccurence, surgical treatment

## Abstract

Plantar fibromatosis, or Ledderhose disease, is a rare benign fibroproliferative disorder of the plantar fascia characterized by the development of fibrous nodules, which may cause pain and functional limitation. Although the exact etiology remains unclear, it has been associated with trauma, diabetes mellitus, liver disease, epilepsy, and chronic alcohol use. Management is often conservative, but surgical intervention may be required when symptoms persist.

A 32-year-old female presented with a six-month history of painful nodules on the medial aspect of her left sole. Clinical examination and ultrasonography revealed three hypoechoic nodules within the plantar fascia. Initial conservative treatment with nonsteroidal anti-inflammatory drugs (NSAIDs), stretching, orthotic insoles, and physiotherapy failed to improve symptoms. Surgical excision with partial plantar fasciectomy was therefore performed. Histopathological examination confirmed the diagnosis of plantar fibromatosis, showing dense fibrocellular tissue with fibrocytic and collagenous proliferation and myofibroblastic differentiation. The postoperative recovery was uneventful, and at five-year follow-up, the patient remained pain-free with no evidence of recurrence. Plantar fibromatosis should be considered in patients presenting with firm nodules on the plantar aspect of the foot. When conservative therapy fails, partial plantar fasciectomy can provide excellent functional outcomes and long-term symptom relief with a low risk of recurrence.

## Introduction

Plantar fibromatosis, also known as Ledderhose disease, is a rare hyperplastic disorder characterized by the development of multiple fibrous nodules within the plantar fascia of the foot. In contrast, a plantar fibroma is a benign nodule that develops on the plantar aspect of the foot. Plantar fibromatosis is an uncommon disorder that most often affects middle-aged individuals, particularly those in their fourth and fifth decades of life. It shows a male predominance, with men affected approximately twice as frequently as women, and bilateral involvement occurs in about one-quarter of cases [1]. The condition is often associated with other fibromatoses, including Dupuytren's and Peyronie’s disease, and its development has been linked to factors such as trauma, liver disease, diabetes mellitus, epilepsy, and chronic alcohol use [2]. A hereditary influence has also been suggested, as cases tend to run in families [1,2]. The nodules typically enlarge slowly and are usually less than 2.5 cm in diameter. More aggressive lesions that grow rapidly and extend in multiple directions are classified as plantar fibromatosis. While some cases remain asymptomatic, others may result in discomfort, pain, and impaired mobility, particularly during walking [2].

The diagnosis of plantar fibromatosis is primarily clinical, relying on palpation of firm nodular masses within the medial and central plantar fascia. Radiographs are not routinely required but may be used to exclude bone involvement. Ultrasound is valuable for detecting nodular lesions within the fascia and assessing changes in surrounding soft tissues, while MRI is particularly useful for identifying deeper extensions and differentiating plantar fibromatosis from other soft tissue disorders [3]. When malignancy is suspected, biopsy with histopathological analysis is performed to rule out sarcomas and other neoplasms. The clinical and imaging appearance of plantar fibromatosis may mimic both benign and malignant soft tissue masses. Common differential diagnoses include subcutaneous fat necrosis, keloids, ganglion cysts, lipomas, desmoid tumors, and foreign-body reactions, as well as malignant lesions such as epithelioid sarcoma, leiomyoma, rhabdomyosarcoma, and liposarcoma. Histological features are often nonspecific; although immunohistochemistry may assist in refining the diagnosis, its role remains limited [4].

Conservative management of plantar fibromatosis focuses on symptom relief and functional improvement through stretching, orthotics, nonsteroidal anti-inflammatory drugs (NSAIDs), physical therapy, and modalities such as transcutaneous electrical nerve stimulation (TENS), deep oscillation therapy, and extracorporeal shock wave therapy (ESWT). Intralesional corticosteroid injections, often ultrasound-guided, may reduce pain and nodule size, although recurrence is common [3]. When conservative measures fail, surgery is indicated, with options including local excision, wide excision, and complete fasciectomy. Recurrence rates are greatest following local excision and lowest after complete fasciectomy, although the latter is the most invasive procedure [3]. We report a rare case of plantar fibromatosis with multiple large nodules that was managed surgically with partial plantar fasciectomy, with no recurrence observed at the five-year follow-up.

## Case presentation

**Figure 1:**
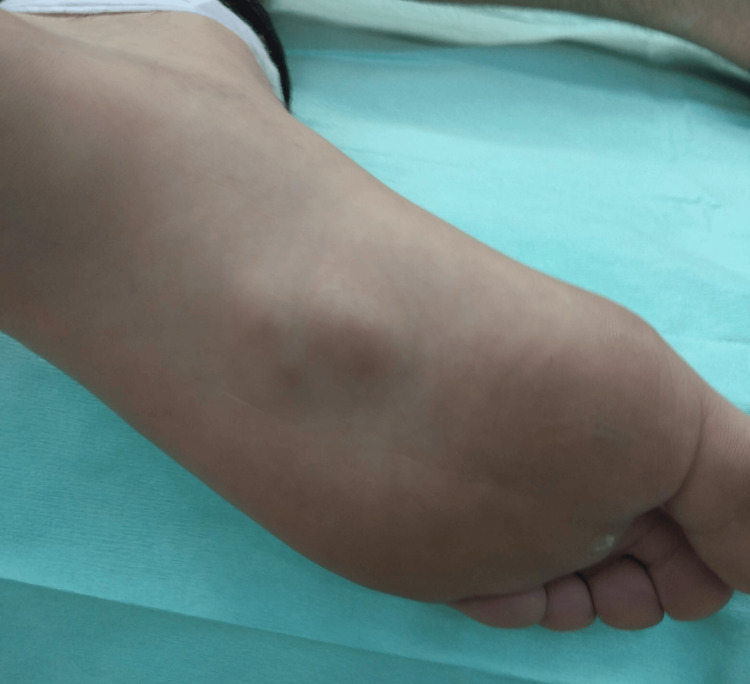
Nodules of different sizes in the middle of the plantar fascia

A 32-year-old female presented to our practice with a six-month history of painful nodules on the medial aspect of her left sole in April 2020. There was no history of previous medical conditions or trauma. She reported plantar pain during walking, which was more pronounced when rising from a seated position, and she also experienced limping during weight-bearing. On examination, three palpable nodules of different sizes were noted approximately in the mid-portion of the plantar fascia. Over the largest nodule, an area of hyperkeratosis was observed (Figure 1).

**Figure 2:**
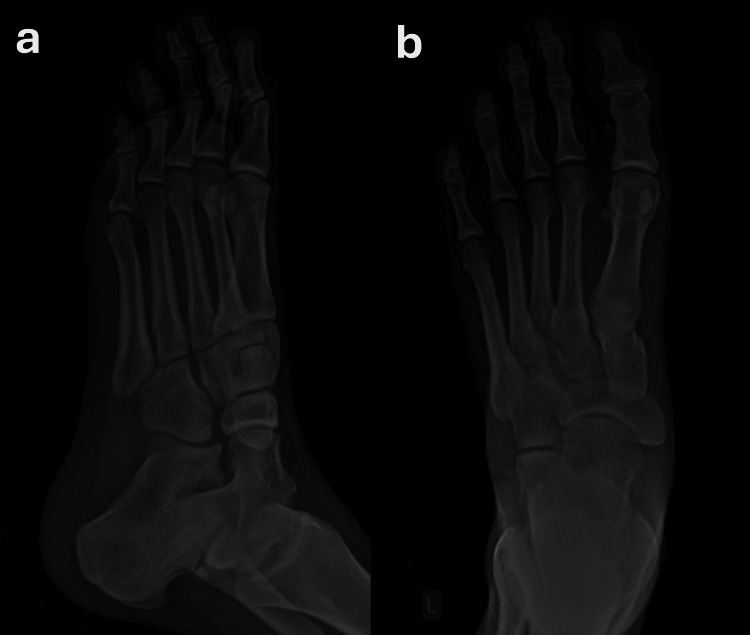
X-ray of the left foot - (a) oblique view; (b) plane view

The nodules were painful on palpation. Additionally, there was tenderness along the entire length of the plantar fascia and restriction in the foot and toe range of motion during passive extension. Examination of the right foot was unremarkable. Plain radiographs of the left foot revealed no calcifications of the soft tissues or other abnormalities of the skeletal structures (Figure 2).

**Figure 3:**
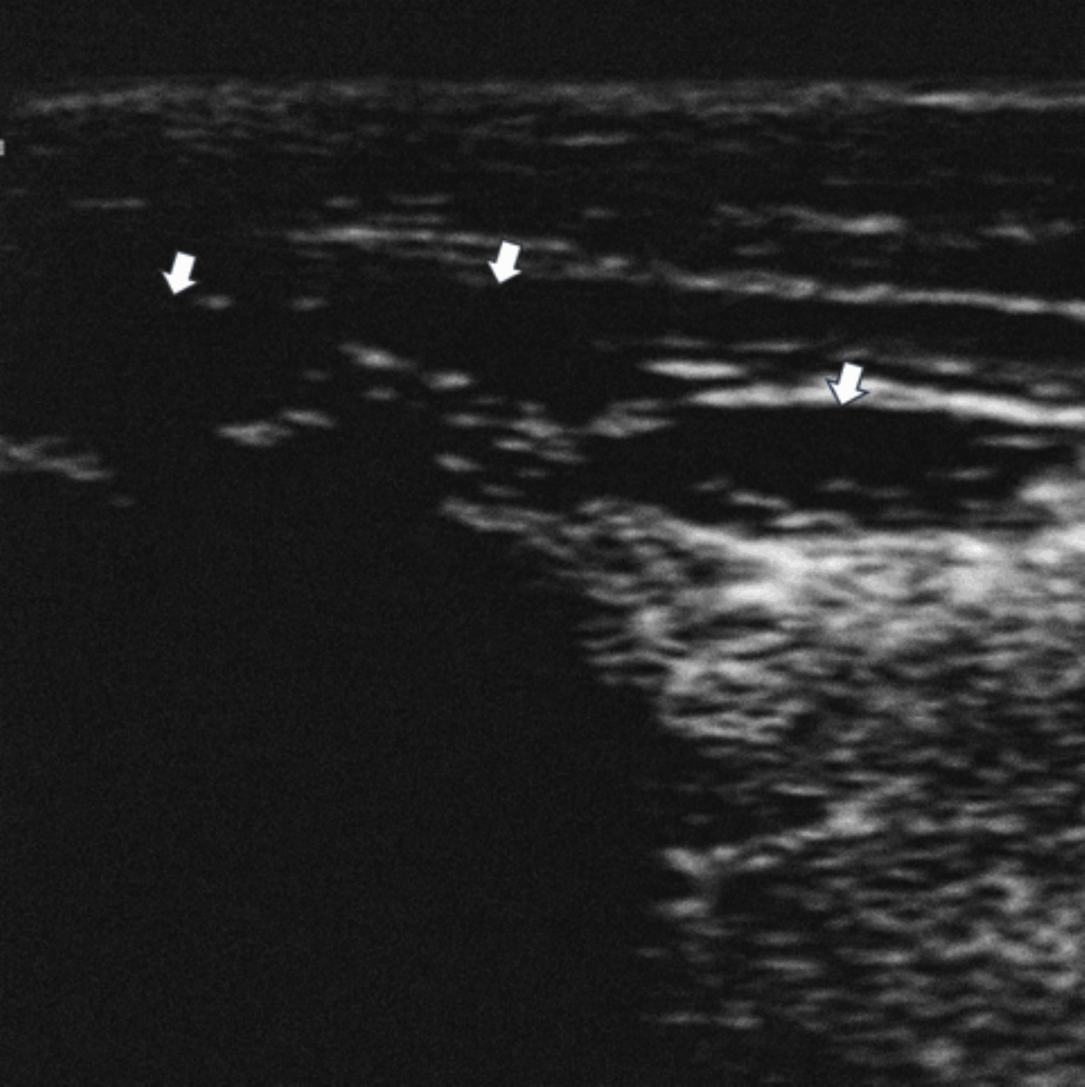
Sonogram of the midfoot showing three differently sized well-defined fusiform hypoechoic nodules (arrows) arising within plantar fascia

Ultrasound examination revealed three hypoechoic, homogeneous nodules of fibrous tissue within the plantar fascia, without internal blood flow on Doppler imaging. The dimensions of the nodules were approximately 2.20 × 0.80 cm, 1.30 × 0.90 cm, and 1.10 × 0.60 cm (Figure 3).

Initial management consisted of NSAIDs (piroxicam 20 mg once daily and paracetamol 1000 mg twice a day), stretching exercises of the plantar aponeurosis, and cryotherapy for 15 days. As there was no improvement, orthotic insoles and physiotherapy were prescribed for the following two months. Physiotherapy included stretching of the aponeurosis, gentle massage and kinesiotherapy, cryotherapy, ESWT, and strengthening exercises of the intrinsic and extrinsic muscles of the foot and leg.

**Figure 4:**
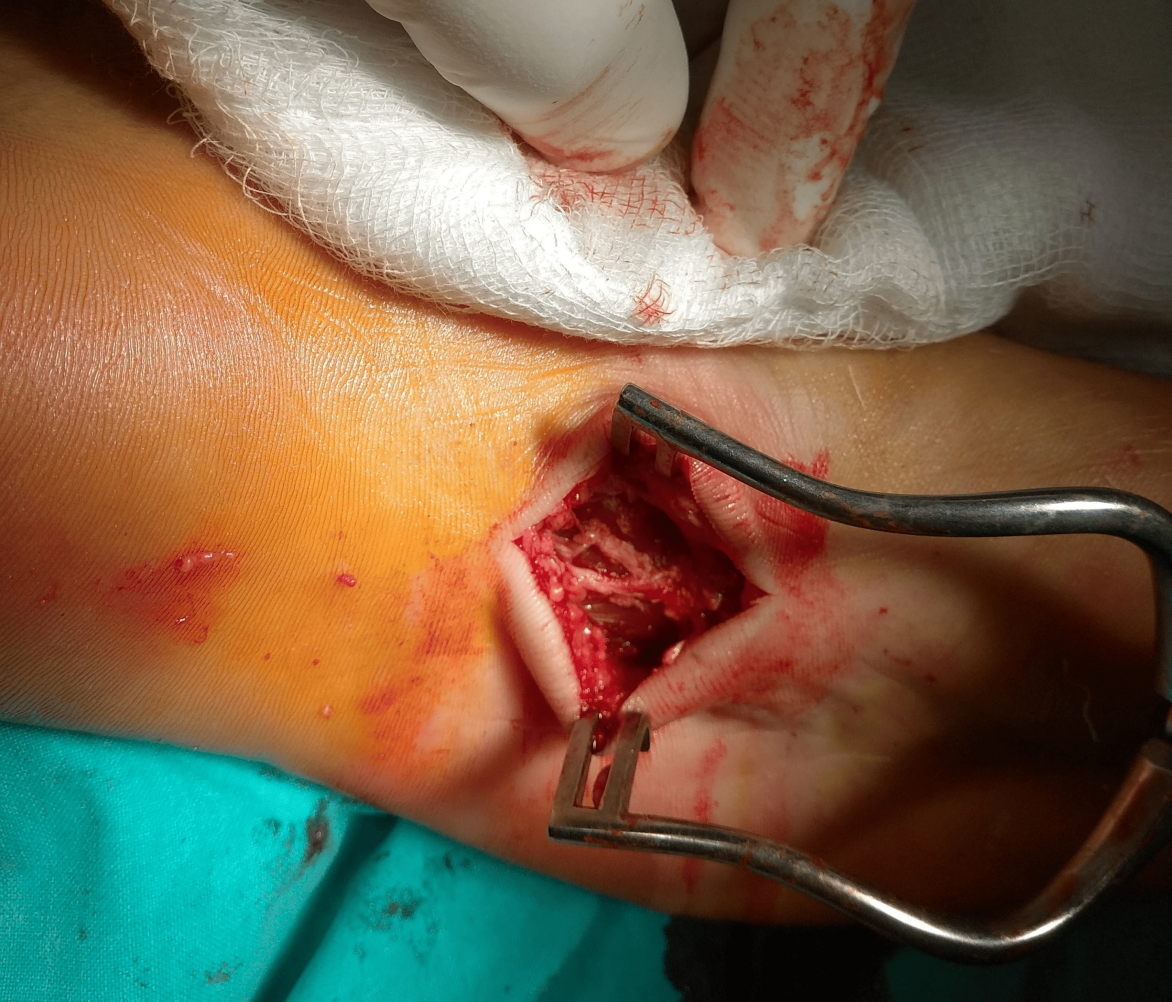
Intraoperative image showing the C-shaped incision and removal of the fibrous nodules with partial fasciectomy

**Figure 5:**
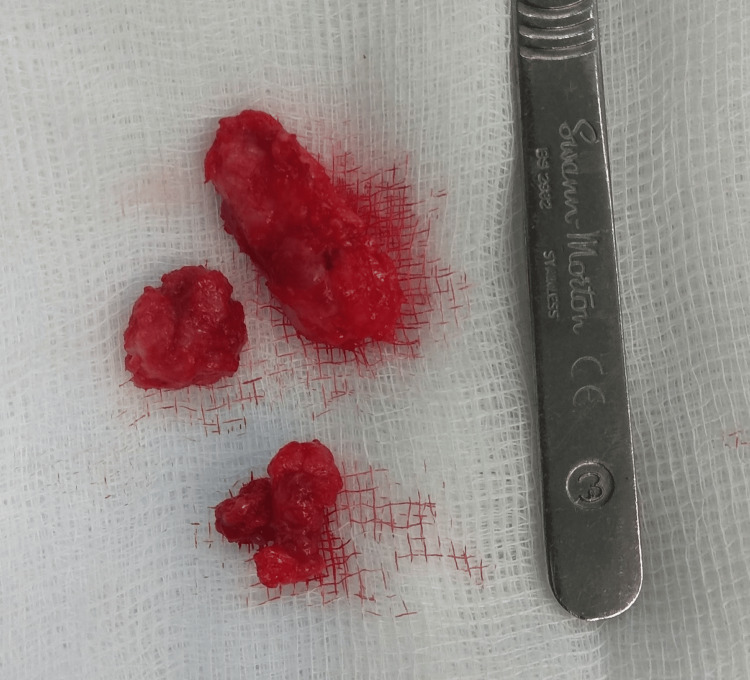
Excised fibrous nodules

After six months of conservative therapy, the patient reported no improvement in symptoms, and her daily activities were significantly impaired. Surgical management was therefore indicated. The patient consented to the procedure and provided written informed consent for publication of this report. Surgical removal of the nodules with partial plantar fasciectomy was performed. A 5-cm C-shaped incision was made medially along the weight-bearing surface. Careful dissection of the skin and subcutaneous tissue was carried out, and the fascia containing the nodules was isolated. The nodules were removed from the underlying muscles and connective tissue, and a partial fasciectomy was completed (Figure 4). The excised tissue was sent for histopathological examination (Figure 5).

**Figure 6:**
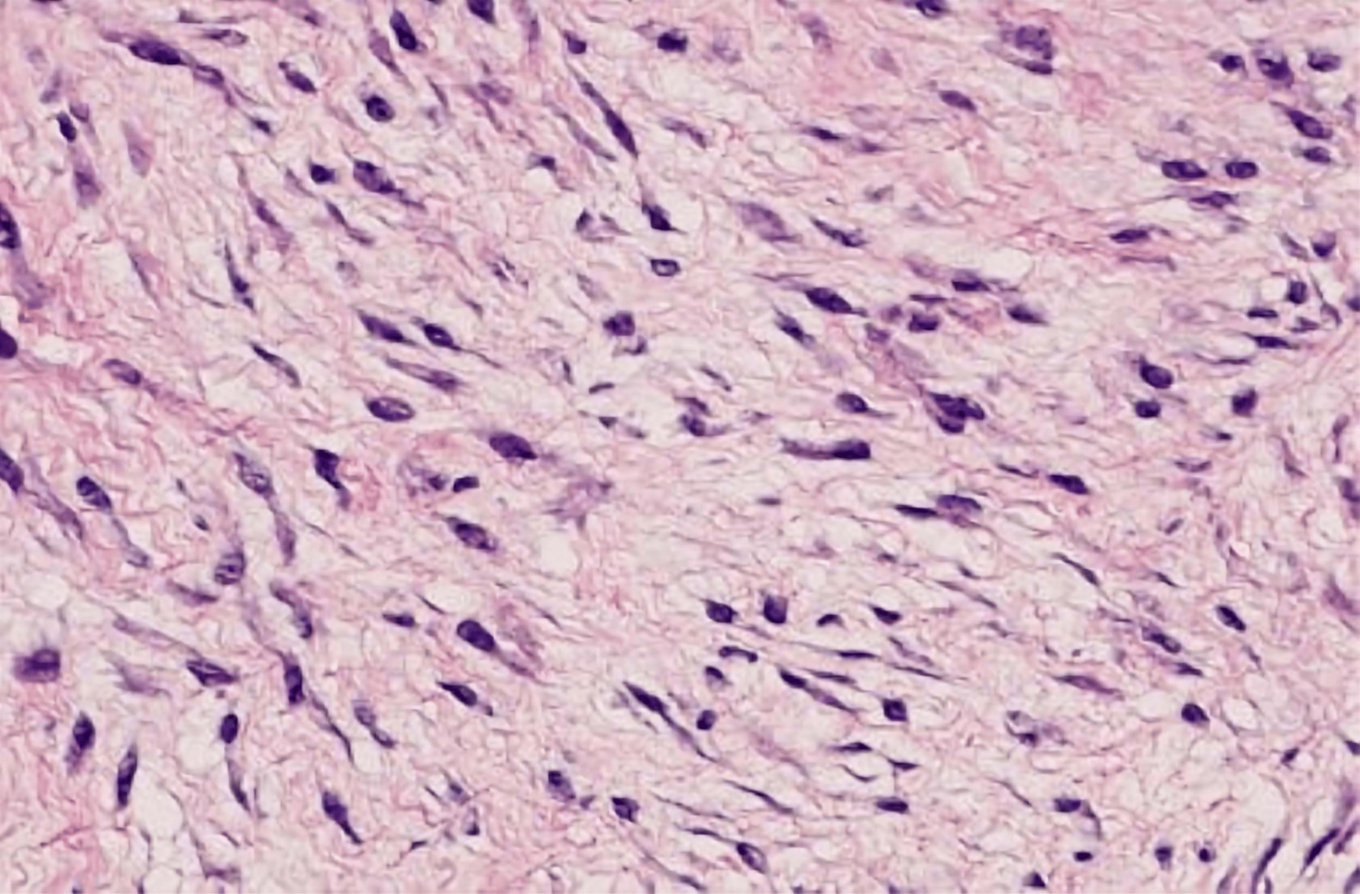
Histological section showing fibrocellular tissue with parallel and nodular arrays of fibrocytes and fibrillar collagen demonstrating corkscrew morphology and myofibroblastic differentiation

Histopathology analysis revealed dense fibrocellular tissue with parallel and nodular arrangements of fibrocytes and fibrillar collagen showing a characteristic corkscrew morphology, with evidence of myofibroblastic differentiation (Figure 6).

The area of hyperkeratosis overlying the largest nodule was also excised. The skin was sutured without tension. The patient was advised to avoid weight-bearing for two weeks, followed by partial weight-bearing for another two weeks. The postoperative period was uneventful. After one month, full weight-bearing in comfortable footwear was allowed, and the patient resumed her daily activities. At three months, she was pain-free, had no limp, and demonstrated a full range of motion. One year postoperatively, she remained completely asymptomatic and had returned to sports activities. At five years of follow-up, there was no evidence of recurrence, and she continued to be asymptomatic with no functional limitations.

## Discussion

Plantar fibromatosis, also known as Ledderhose disease, is a rare benign fibroproliferative disorder of the plantar fascia, first described by Georg Ledderhose in 1897. It is characterized by fibrous tissue overgrowth and the formation of nodules, typically located in the midfoot or forefoot region [5]. Its exact etiology remains unclear; however, it has been associated with repetitive microtrauma, genetic predisposition, chronic phenobarbital use, alcohol abuse, diabetes mellitus, epilepsy, smoking, and related fibromatoses such as Dupuytren’s and Peyronie’s diseases [1,6]. A hereditary influence has also been suggested, as cases tend to run in families [1,2]. The condition typically arises in middle-aged individuals, most often in their forties and fifties. It is more common in men, who are affected roughly twice as frequently as women, and both feet are involved in about one-quarter of cases [1]. 

Ledderhose disease progresses through three histopathological phases. In the proliferative phase, multinodular expansion of immature spindle cells with minimal collagen is observed [7]. The intermediate phase is characterized by active fibroblast proliferation and the initial formation of collagen bundles [8]. In the residual phase, the lesions become less cellular and more collagenized, occasionally leading to contractures or flexor deformities [7]. Fibromas usually become clinically evident in the later portions of phases II and III. Two subtypes exist: juvenile fibromatosis, which tends to be aggressive and infiltrative, and adult-type fibromatosis, which progresses more slowly and rarely invades adjacent structures. Plantar fibromatosis can extend into the intrinsic foot muscles, flexor digitorum brevis, and abductor hallucis tendons, and may involve the medial and lateral plantar nerves and adjacent vascular networks [1].

The diagnosis of plantar fibromatosis is primarily clinical, supported by the patient’s history and physical examination, with imaging and biopsy used for confirmation when necessary [9]. Physical evaluation should include inspection for swelling, skin changes, and deformities; palpation of the plantar fascia; and assessment of range of motion and gait. Concomitant contractures of the Achilles tendon or gastrocnemius should also be documented, as these may exacerbate symptoms [10]. Plantar fibromas are typically palpable nodules along the medial plantar fascia, sometimes associated with toe flexion contractures, particularly of the hallux [10].

Imaging aids in confirming the diagnosis, defining the extent of fibromatous involvement, and excluding other plantar pathologies [11]. While radiographs are often non-diagnostic, MRI and ultrasound provide superior soft-tissue characterization. MRI typically reveals a focal or lobulated mass within the plantar fascia, demonstrating low signal intensity on T1-weighted and low-to-intermediate intensity on T2-weighted sequences. Low T2 signal generally corresponds to inactive or residual disease, while intermediate signal suggests active fibroblastic proliferation [9]. Ultrasound is often preferred as a first-line modality due to its accessibility and dynamic assessment capability, enabling detailed evaluation of lesion size, number, and fascial depth [12].

The differential diagnosis includes plantar fasciitis, tarsal tunnel syndrome, calcaneal fracture, and soft-tissue neoplasms such as leiomyoma, liposarcoma, neurofibroma, rhabdomyosarcoma, fibrosarcoma, and nodular fasciitis [1]. Other benign conditions that can mimic plantar fibromatosis include ganglion cysts, keloids, lipomas, and epidermal inclusion cysts [11]. Histopathological examination is essential when malignancy is suspected, as it helps differentiate fibromas from sarcomatous lesions and guides appropriate treatment [12].

In the early stages, management is conservative since most lesions progress slowly and often respond to non-surgical measures [8]. The main goals are to relieve pain, maintain foot function, and limit disease progression rather than achieve complete regression [1]. Conservative modalities include NSAIDs, activity modification, and physiotherapy aimed at improving mobility and strength [13]. Orthotic devices and rocker-bottom footwear may reduce pressure and strain across the plantar fascia. Adjunctive therapies such as TENS and deep oscillation therapy may improve local circulation and pain control [14]. Ultrasound-guided corticosteroid injections can reduce pain and nodule size by modulating fibroblast activity, although recurrence is frequent [15]. ESWT has also demonstrated efficacy in pain reduction and nodule softening [16]. Other experimental therapies, including collagenase, verapamil, tamoxifen, and radiotherapy, have shown variable outcomes in refractory cases [17].

Surgery is reserved for patients with persistent pain or functional limitation despite adequate conservative treatment. The surgical goal is complete excision of the lesion with a margin of macroscopically normal aponeurosis; adherent overlying skin should also be removed if necessary [18]. Options include local excision, wide excision, and complete fasciectomy. Local excision is associated with the highest recurrence rate and is rarely recommended as a primary approach. Wide excision, involving removal of the lesion with surrounding fascia, may reduce recurrence risk, though outcomes remain variable [17]. Complete fasciectomy, involving extensive removal of the plantar aponeurosis, offers the lowest recurrence rate but entails higher morbidity [19]. Since the fibromatous tissue is infiltrative and non-encapsulated, limited resections often leave microscopic remnants, predisposing to recurrence [19].

Recurrence may occur regardless of treatment. Recurrence after surgical removal of plantar fibromatosis remains high across all techniques, with local excision carrying the highest risk, consistently reported between 57% and 100% [3,5,12,17]. Wider excision, which includes a 2-3 cm margin of surrounding tissue, demonstrates more variable but generally lower recurrence rates, ranging from 8 to 80% [3,17]. The lowest recurrence rates are observed with partial or complete fasciectomy, reported between 0% and 50%, making it the most effective option for minimizing recurrence despite being the most invasive [3,12,17]. Risk factors include multiple nodules, bilateral disease, early onset, and a history of prior regrowth [18,20]. Careful incision planning is critical, as fasciectomy carries risks of wound healing complications, painful scarring, and nerve entrapment. Longitudinal or lazy “S” incisions along the medial arch are preferred to preserve vascular integrity [17]. Postoperatively, patients are advised to remain non-weight-bearing for about three weeks, followed by gradual rehabilitation until full recovery.

## Conclusions

In this case, partial plantar fasciectomy successfully relieved the patient’s pain and restored full foot function, with no recurrence observed over a five-year follow-up, demonstrating the efficacy of surgical excision for multiple symptomatic plantar nodules. This outcome highlights that, when conservative therapy fails, carefully planned partial fasciectomy can provide durable symptom relief. More broadly, plantar fibromatosis should be considered in patients with firm plantar nodules, and individualized management strategies are essential for optimal long-term results.
